# Host specificity and uniqueness of shell microbiome in freshwater mollusks

**DOI:** 10.3389/fmicb.2025.1702047

**Published:** 2025-12-15

**Authors:** Zifan Zhao, Zhendu Mao, Dan He, Heng Wang, Qinglong Wu

**Affiliations:** 1 Center for Evolution and Conservation Biology, Southern Marine Science and Engineering Guangdong Laboratory (Guangzhou), Guangzhou, China; 2 Key Laboratory of Lake and Watershed Science for Water Security, Nanjing Institute of Geography and Limnology, Chinese Academy of Sciences, Nanjing, China; 3 Sino-Danish Center for Science and Education, University of Chinese Academy of Sciences, Beijing, China; 4 Fuxianhu Station of Deep Lake Research, Chinese Academy of Sciences, Chengjiang, Yunnan, China

**Keywords:** lake, mollusk, snail, clam, shell microbiome

## Abstract

**Introduction:**

Mollusk shells represent a major substrate for the colonization of microbial communities and the functioning of aquatic ecosystems. However, our knowledge of the shell microbiome is very limited.

**Methods:**

Here, we selected *Bellamya aeruginosa* and *Corbicula fluminea* as two types of snails and clams, respectively, to explore their shell epiphytic bacteria by 16S amplicon sequencing.

**Results:**

We found different shell bacterial communities between snails and clams, which were also distinct from those in the surrounding environment. Source tracking analysis indicated that snail-shell bacteria were mostly derived from sediments, whereas clamshells originated from tissues. There was a site-specific difference in the shell bacteria within the habitat. Temporal variation in clamshell bacteria was observed, but not in snail shells, which corresponds to their source dynamics in the water column and stable surface sediment bacterial communities, respectively. The genus *Nitrospira* is mostly enriched in shell bacteria, particularly in eutrophic lakes. Taxa related to carbon, nitrogen, and sulfur cycling were recognized as the keystone species in the co-occurrence network associated with the shell surface. Our results demonstrate that mollusk shells represent a unique ecological niche for microbiomes in aquatic ecosystems and may serve as hotspots for biogeochemical cycling.

## Introduction

With increasing attention given to host-associated microbiota, recent studies have suggested that it not only plays important roles in host development, immunity, metabolism, behavior, and numerous other processes but also carries out biogeochemical transformations in its living environment (Petersen et al., 2016; Mallott and Amato, 2021; Luan et al., 2023; Wainwright et al., 2023; Krishnaveni et al., 2025). However, most studies still focus on microorganisms occurring inside tissues/organs. The surfaces of aquatic animals also provide a special natural substrate for microbial colonization, life activity, and evolution (Kuschke, 2022). Host surface-associated microbiota are still rarely studied in aquatic animals, despite their importance in both host and ecosystem ecology.

Host-associated bacteria are tissue-specific, and their assembly is dominated by a deterministic process related to the host genotype and life history (Mallott and Amato, 2021; Sadeghi et al., 2023). A meta-analysis study based on 654 host species and over 15,000 samples revealed that internal microbiomes are best explained by host factors, such as phylogeny/immune complexity and trophic level/diet, along with climate, whereas host surface-associated microbiomes are more strongly influenced by environmental factors (Woodhams et al., 2020). In aquatic ecosystems, different species of marine macroalgae and sponges in the same habitat support different epibiotic bacterial communities, whereas the same algal or animal species from different environments or locations tend to maintain relatively consistent epibiotic communities (Malik et al., 2020; Williams et al., 2024; Liu et al., 2025). Mollusk shells, composed of over 96% calcium carbonate with high alkalinity for the pH buffer, provide unique sites for the attachment and growth of microorganisms (Yoon et al., 2003; Liu et al., 2010). Previous research on oysters has demonstrated that shell-associated bacterial communities differ significantly from both internal host microbiota and environmental microbial assemblages (Dubé et al., 2019; King et al., 2021). However, despite their ecological importance, the composition, assembly mechanisms, functional roles, and spatiotemporal dynamics of mollusk shell microbiota remain poorly understood. Further investigation is required to elucidate the drivers that shape these microbial communities and their ecological significance in aquatic ecosystems.

Microbial communities colonizing abiotic surfaces (e.g., soil, rock, wood, glass, and plastic in aquatic ecosystems) play crucial roles in various ecological processes, including nitrogen fixation, nitrification, carbon sequestration, pollutant adsorption, pathogen transmission, and the dissemination of antibiotic resistance genes (Wang et al., 2017; Bhagwat et al., 2021; Meng et al., 2023; Liu et al., 2024; Zhou et al., 2024). Recently, microbial colonization of artificial substrates, particularly microplastics, has emerged as a significant research focus (Qian et al., 2022). It has been proven that plastisphere biofilms represent a novel nitrifying niche in estuarine ecosystems, exhibiting distinct biogeochemical characteristics compared to their planktonic counterparts (Su et al., 2024). Similarly, the shell microbiota of bivalves has been shown to participate extensively in nitrogen cycling, with both nitrification and denitrification occurring in their shell biofilms because of heterogeneous oxygen distribution (Stief et al., 2009; Svenningsen et al., 2012; Caffrey et al., 2016). Despite these findings, our understanding of the broader ecological functions of host-associated shell microbiota remains limited.

*Bellamya aeruginosa* and *Corbicula fluminea* are representative snails and clams, respectively, in Asian freshwater ecosystems, where they often dominate shallow water habitats and achieve high population densities (Yao et al., 2020; Wang et al., 2023). In this study, two mollusks were selected to investigate the epibiotic bacterial communities associated with shells. *B. aeruginosa* is a gastropod snail with a herbivorous deposit-feeding lifestyle that preferentially inhabits areas containing submerged macrophytes and organic-rich soft sediments (Ma et al., 2017). *C. fluminea* is a filter-feeding bivalve clam that frequently dominates eutrophic systems because of the high availability of particulate organic matter (Modesto et al., 2025). Notably, *C. fluminea* has emerged as one of the most invasive freshwater bivalves in recent decades, expanding its range from Asia to Europe, North America, and Africa (Crespo et al., 2015). Despite their ecological significance, the shell-associated bacterial communities of these two species remain uncharacterized. In this study, mollusk samples were collected from Lake Taihu, a large shallow lake where these two mollusks dominate benthic mollusks. The bacterial communities of 167 samples associated with the tissue and shell of two mollusks, particle-associated and sediment, across 3 seasons and 3 sampling sites in Lake Taihu were investigated by means of 16S rRNA gene sequencing. We aimed to characterize the diversity and community structure of the bacteria associated with the shells of the two mollusks. In particular, we aimed to determine whether there are host-specific differences in shell bacterial composition and the potential mechanisms underlying these differences.

## Materials and methods

### Sample collection and preparation

**Figure 1:**
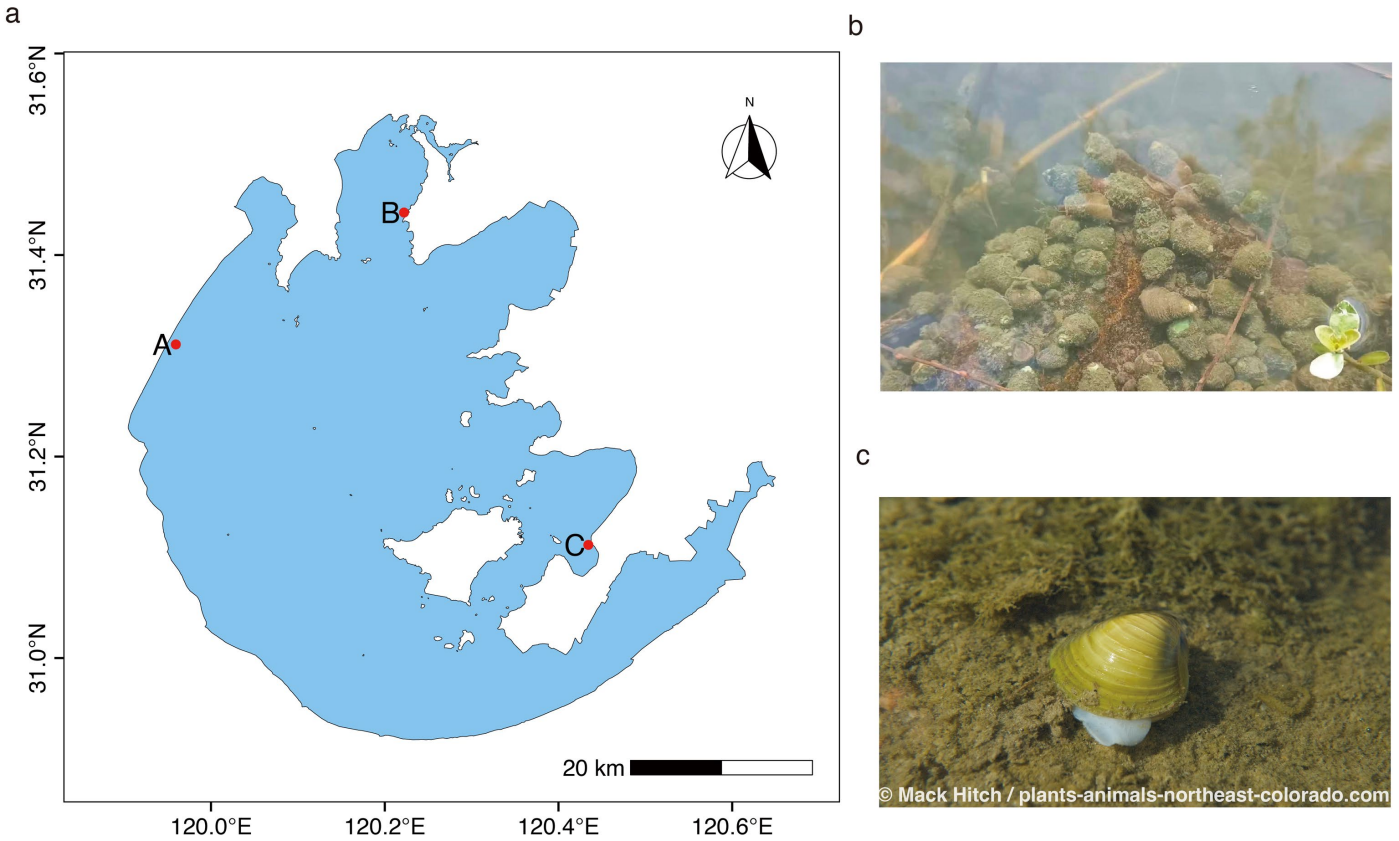
**(a)** Sampling sites in Lake Taihu, China. The red dots indicate the three sampling sites. Photography of the **(b)** snail and **(c)** clam. The clam image was sourced from plants-animals-northeast-colorado.com and copyrighted by Mack Hitch.

Water, sediment, snail *B. aeruginosa* (later abbreviated as snail), and clam *C. fluminea* (later abbreviated as clam) were collected from three sampling sites across Lake Taihu in October 2020, January 2021, and May 2021. Taihu Lake (30°55′40″ to 31°32′58”N, 119°52′32″ to 120°36′91″E) is a typical large, shallow eutrophic lake in China with an area of 2,427.8 km^2^ and an average depth of 1.9 m. Sampling sites A (31°18′52”N, 119°56′42″E) and B (31°27′5”N, 120°12′18″E) are located in the most eutrophic part of the lake, which exhibits a cyanobacterium-dominated turbid state for most of the year; site C (31°07′33”N, 120°25′39″E) is located in the submerged macrophyte-dominated region, which remains in a clear-water state (Qin et al., 2007) (Figure 1). Surface water samples were collected at a depth of 0.5 m with a 5-L Schindler sampler. Snail, clam, and sediment (0–10 cm) samples were collected using a Peterson grab sampler (1/16 m^2^) at each sampling site. All collected samples were transported to the laboratory for further processing within 3–6 h. Three replicated samples of water and sediment were taken at each sampling site and season. During most sampling sites and seasons, three clams and snails were collected. Two or no individuals were sampled at some sites/seasons. In total, 23 snail and 22 clamshell samples, 18 snail and 23 clam tissue samples, 27 samples of particle-associated bacteria, 27 samples of free-living bacteria, and 27 samples of sediment bacteria were collected. The detailed sample numbers can be found in Supplementary Table S1.

Water temperature (T), dissolved oxygen (DO), pH, and conductivity (Cond) were measured *in situ* using a multiparameter water quality probe (YSI 6600, Yellow Springs, OH, USA). Turbidity (NTU), total nitrogen (TN), total phosphorus (TP), and chlorophyll a (Chl-a) were analyzed according to standard methods (State Environmental Protection Administration of China, 2002). The physicochemical characteristics of the water column were summarized in Supplementary Table S2.

Approximately 500 mL of water from each sampling site was filtered sequentially with 5-mm and 0.22-mm filters (Isopore membrane filters; Merck Millipore) for analyses of the diversity of particle-associated and free-living bacteria, respectively. All of the filter membranes were stored at −80 °C until microbial DNA extraction. We rinsed each zoobenthos individual with sterile water to remove sediment and transient bacteria, then swabbed the shell using a different sterile rayon-tipped swab (Copan, 3510C, Italy) for each individual. Each zoobenthos individual was stored overnight for about 10 h in a 100-mL transparent plastic bottle with sterile water to evacuate the gut contents. Soft tissue of the clam and snail was dissected using sterile knives and scissors as a whole, and after rinsing with sterile water several times, the soft tissue was placed into a 1.5-mL tube for further treatments. The shell and tissue samples were processed individually prior to DNA extraction. Sediment and tissue samples were freeze-dried and stored at −80 °C until microbial DNA extraction.

### DNA extraction and high-throughput sequencing

DNA extraction and high-throughput sequencing. DNA was extracted from the filter membranes, sediments, swabs, and tissues using the DNeasy PowerSoil Pro DNA isolation kit (Qiagen, Germanton, MD, USA) according to the manufacturer’s instructions. The soft tissue was homogenized in the lysis buffer provided with the DNA isolation kit. Primers 515F (59-GTGYCAGCMGCCGCGGTAA-39) and 806R (59-GGACTACNVGGGTWTCTAAT-39) targeting the V4 region of bacterial 16S rRNA genes were selected for PCR amplification (Caporaso et al., 2010). The PCR protocol consisted of an initial denaturation step at 95 °C for 2 min followed by 25 cycles of denaturation at 95 °C for 30 s, annealing at 55 °C for 30 s, and elongation at 72 °C for 30 s, with a final extension step at 72 °C for 5 min. Each reaction mixture contained 4 mL of 5 × FastPfu buffer, 2 mL of 2.5 mM deoxynucleoside triphosphates (dNTPs), 0.8 mL each of 5 mM forward and reverse primers, 0.4 mL of FastPfu polymerase, and 10 ng of template DNA. Triplicate PCR products were pooled, purified, and used for library construction according to Illumina’s genomic DNA library preparation procedure and sequenced using the Illumina MiSeq platform (Shanghai Biozeron). Negative controls for DNA extraction and PCR amplification were also performed to exclude potential contamination. We did not find any potential contamination for the negative controls. All the samples were sequenced successfully and considered for downstream analysis.

Raw fastq files were first demultiplexed using Trimmomatic (Bolger et al., 2014)^1^ and in-house Perl scripts according to the barcode sequences information for each sample with the following criteria: (i) The 300 bp reads were truncated at any site receiving an average quality score <20 over a 10 bp sliding window, discarding the truncated reads that were shorter than 50 bp. (ii) exact barcode matching, 2 nucleotide mismatches in primer matching, and reads containing ambiguous characters were removed. (iii) only sequences that overlap longer than 10 bp were assembled according to their overlap sequence. Reads that could not be assembled were discarded. Passed sequences were dereplicated and subjected to the DADA2 algorithm (QIIME 2 recommended) to identify indel mutations and substitutions (Edgar, 2010). The trimming and filtering were performed on paired reads with a maximum of two expected errors per read (maxEE = 2). After merging paired reads and chimera filtering, the phylogenetic affiliation of each 16S rRNA gene sequence (herein called ASVs) was analyzed by the UCLUST algorithm^2^ against the Silva (SSU138.1) (Quast et al., 2013) 16S rRNA database^3^ using a confidence threshold of 80% (Kim, 2014). All samples were randomly subsampled to 21,407 reads prior to downstream analysis. Alpha diversity was calculated using the vegan and picante packages in R (Dixon, 2003; Kembel et al., 2010).

### Statistical analysis

Fast expectation maximization for microbial source tracking (FEAST) was used to predict the contributions of the surrounding environment and tissue to the microbiota of individual zoobenthos shells at the genus level using the FEAST R package (Shenhav et al., 2019). Bacterial functional profiles were predicted using functional annotation of prokaryotic taxa (FAPROTAX) (Louca et al., 2016). Functional differentiation of the bacterial communities in shell and environmental samples was performed, and graphics were generated using STAMP (v2.1.3) through Welch’s *t*-test, and *p* values were adjusted with the Benjamini-Hochberg false discovery rate (FDR) multiple-test correction (Parks et al., 2014). Bray–Curtis dissimilarity matrices were used for non-metric multidimensional scaling (NMDS) to assess differences in beta diversity by PERMANOVA (permutational multivariate analysis of variance), using the adonis function of the vegan R package. The null-model analysis was applied to evaluate the assembly processes (deterministic or stochastic) of bacterial communities associated with the shell of zoobenthos. We calculated the beta nearest taxon index (βNTI) using the picante R package. βNTI values between −2 and +2 indicate the dominance of stochastic processes, while βNTI values above +2 or below −2 indicate the dominance of deterministic processes. Non-parametric statistical tests were run to evaluate alpha diversity, source contributions, and relative abundance differences across groups, using the *wilcox.test* and *kruskal.test* functions of the *stats* R package. Graphs were generated using the *ggplot2* R package.

### Microbial network analysis

The 500 most abundant ASVs were kept for network analysis to avoid introducing spurious correlations. The network analysis was conducted using the ggClusterNet package in R by selecting Spearman correlation measures, and the inferred correlations were restricted to those having correlations greater than 0.75 or less than −0.75 (*p* = 0.05) (Wen et al., 2022). Nodes in a modularized network could be classified as follows: (i) peripheral nodes (*Z* ≤ 2.5, *p* ≤ 0.62, nodes having few links to other nodes within and outside their modules); (ii) connectors (*Z* ≤ 2.5, *p* > 0.62, nodes highly connected to several modules); (iii) module hubs (*Z* > 2.5, *p* ≤ 0.62, nodes highly connected to other nodes within their own modules); and (iv) network hubs (*Z* > 2.5, *p* > 0.62, nodes act as both module hubs and connectors) (Guimerà and Nunes Amaral, 2005; Zhou et al., 2011).

### Data availability

Raw data files have been made available at the NCBI Sequence Read Archive under BioProject accession number PRJNA1212329.

## Results

### Diversity and community composition of bacteria associated with shells

**Figure 2:**
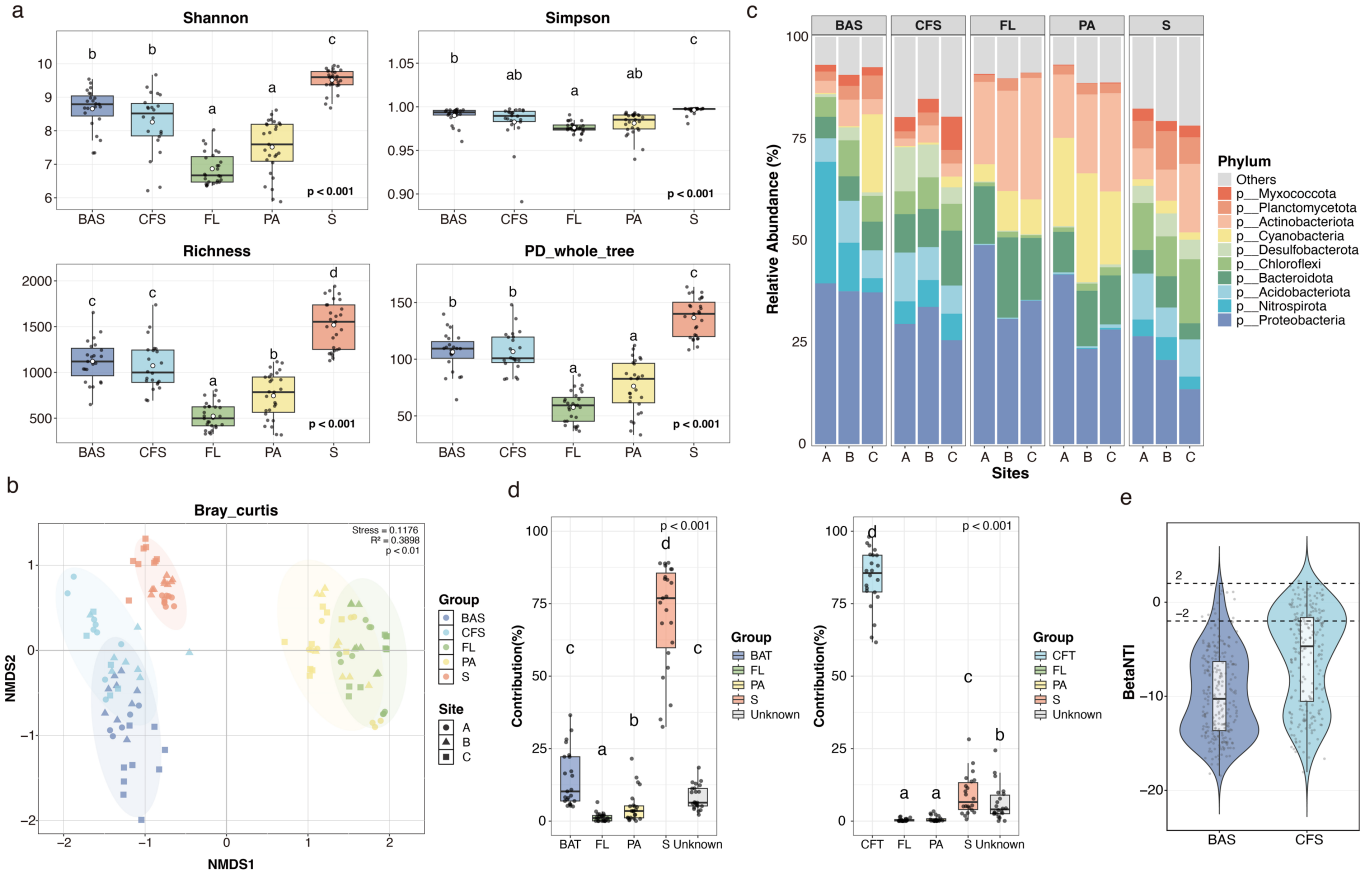
Comparison of the microbial communities in *Bellamya aeruginosa* shell (BAS), *Corbicula fluminea* shell (CFS), free-living (FL), and particle-associated (PA) bacterioplankton and sediment (S) samples. **(a)** Alpha diversity: Shannon index, Simpson index, richness, and phylogenetic diversity. **(b)** Beta diversity: Non-metric multidimensional scaling (NMDS) analysis based on Bray–Curtis dissimilarity. **(c)** Relative abundance and taxonomic composition of microbiota in the BAS, CFS, FL, PA, and S samples at the phylum level. A, B, and C indicate the sampling sites. **(d)** FEAST analysis depicting the contributions of microbiota from FL, PA, S, and *B. aeruginosa* tissue (BAT) or *C. fluminea* tissue (CFT) to the shell microbiota. The boxes in (a,d) represent the upper and lower quartiles, the horizontal lines indicate the medians, the whiskers show the 95% range, and the points are outliers. The letters above the columns indicate significant differences between different groups (two-sided Wilcoxon rank-sum tests with Benjamini–Hochberg correction, *p* < 0.001, 95% CI). **(e)** Effects of deterministic (|βNTI | ≥ 2) and stochastic (|βNTI | < 2) processes in shaping *B. aeruginosa* shell (BAS) and *C. fluminea* shell (CFS)-associated bacterial communities. (BAS = 23, CFS = 22, BAT = 18, CFT = 23, FL = 27, PA = 27, and S = 27).

The Shannon, Simpson, and ASV richness and phylogenetic diversity in samples of snail shell (8.66 ± 0.127, 0.99 ± 0.002, 1,121 ± 47.7, 106 ± 3.58) and clamshell (9.51 ± 0.064, 0.996 ± 0.001, 1,518 ± 50.5, 137 ± 3.25) were significantly lower than in sediment (8.26 ± 0.195, 0.983 ± 0.005, 1,073 ± 57.1, 107 ± 3.78) and higher than in free-living (6.87 ± 0.156, 0.976 ± 0.001, 522 ± 26.8, 57.5 ± 2.64) and particle-associated bacteria (7.51 ± 0.156, 0.981 ± 0.003, 745 ± 47.6, 76.2 ± 4.37) (*p* < 0.001 for all) (Figure 2a). Non-metric multidimensional scaling (NMDS) ordination using Bray–Curtis dissimilarity matrices revealed that microbial communities clustered by sample type (i.e., snail shell, clamshell, free-living, particle-associated, and sediment), and the bacterial community associated with shells was significantly different from surrounding environmental samples (*R*^2^ = 0.3898, *p* = 0.01), which most resembled that of sediment bacteria samples (Figure 2b). In total, about 9,308, 9,815, 5,262, 8,755, and 15,491 ASVs were found in snail shell, clamshell, free-living, particle-associated, and sediment samples, respectively. 4,572 and 4,551 of the ASVs were only found in snail shells and clamshells, which accounted for 49.1 and 46.4% of their total ASVs (Supplementary Figure S1).

**Figure 3:**
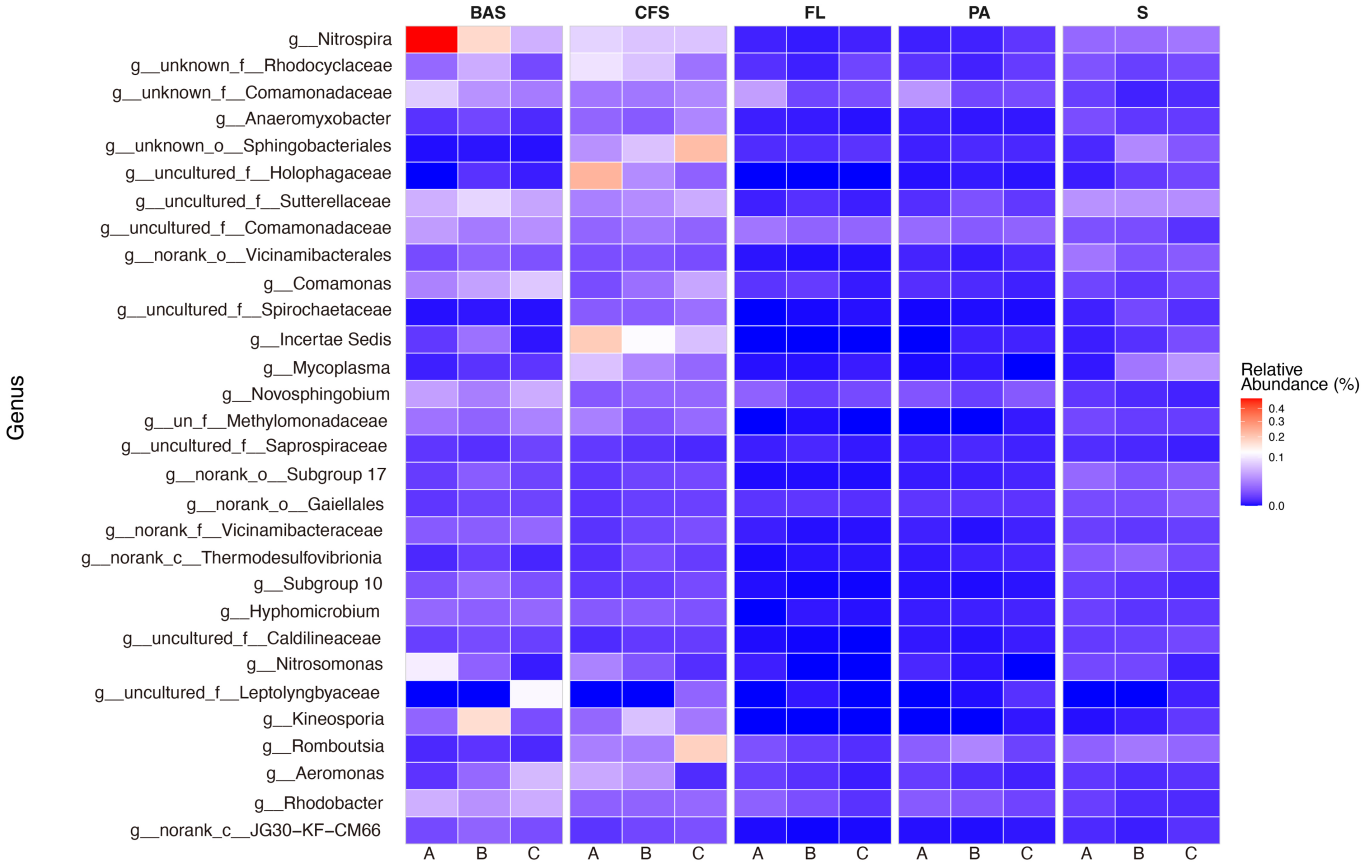
Heat map of the top 30 genera in the *B. aeruginosa* shell (BAS), *C. fluminea* shell (CFS), free-living (FL), and particle-associated (PA) bacterioplankton and sediment (S) samples. A, B, and C indicate the sampling sites. (BAS = 23, CFS = 22, FL = 27, PA = 27, and S = 27).

There was a significant difference in shell bacterial communities between snail and clam (*R*^2^ = 0.1578, *p* = 0.001). The most abundant phyla (≥ 10%) of snail shells were Proteobacteria and Nitrospirota, with averages of 37.8 and 12.5%, while clamshells were enriched with Proteobacteria and Bacteroidota, with averages of 30.1 and 10.3% (Figure 2C). The relative abundance of *Proteobacteria, Actinobacteriota, Cyanobacteria, Nitrospirota,* and *Planctomycetota* in the *B. aeruginosa* shell was greater than in the clamshell, and *Bacteroidota, Myxococcota,* and *Desulfobacterota* were significantly abundant in the clamshell (Supplementary Figure S2). The top 30 genera of shell bacteria are shown in Figure 3. Interestingly, *Nitrospira* is found to be the most abundant genus in both snail and clamshell bacterial communities.

Fast expectation maximization for microbial source tracking (FEAST) was used to identify potential sources of microbes associated with the shell. All of the environmental samples and tissue samples were pooled as potential sources. The source contribution of bacteria associated with snail shells was mostly from sediment (70.9% ± 18.2%), followed by snail tissue (14.5 ± 9.62%), particle-associated bacteria (5.02 ± 5.56%), and free-living bacteria (1.33 ± 1.54%). However, the clam tissue (83.6 ± 10.2%) sample is a significant contributor to shell bacterial communities of the clam, followed by sediment bacteria (9.03 ± 6.90%), particle-associated bacterial sample (0.723 ± 1.02%), and free-living bacteria (0.377 ± 0.476%) (Figure 2d).

### Intra-habitat and seasonal differences among the shell bacteria

**Figure 4:**
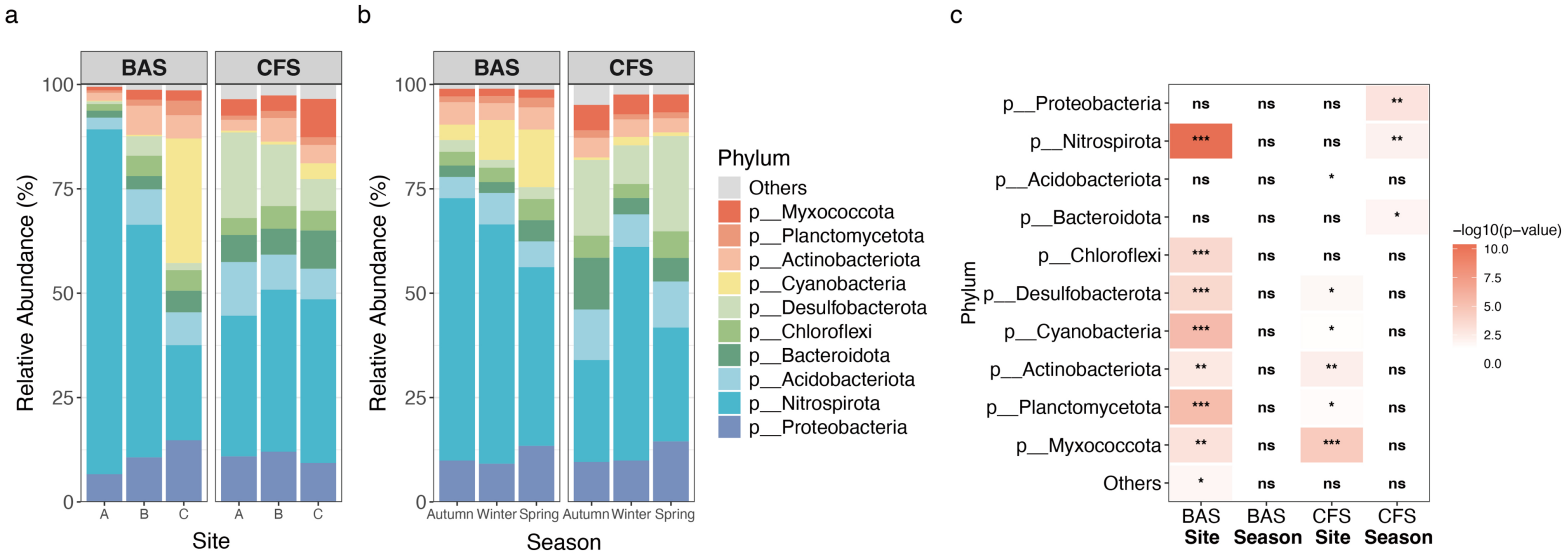
Relative abundance of bacteria associated with **(a)** *B. aeruginosa* shell (BAS) and **(b)** *C. fluminea* (CFS) shell samples at the phylum level. **(c)** Statistical significance of site and season differences in the top phyla of bacteria associated with BAS and CFS. *** Indicates *p* < 0.001, ** indicates *p* < 0.01, * indicates *p* < 0.05, and ns indicates not significant (paired two-sided Wilcoxon rank sum test, 95% CI; BAS = 23, CFS = 22).

PERMANOVA revealed that the bacteria present in shells from different sampling sites were distinct from each other (R^2^ = 0.5089 for snails and R^2^ = 0.2842 for clams, *p* = 0.001) (Table 1 and Supplementary Figure S3). However, a season-specific difference was found only in the clamshell samples (*R* = 0.2009, *p* = 0.001) (Table 1 and Supplementary Figure S3). The main phyla showed significant differences across the seasons (Figure 4c). Site differences were observed in all types of environmental samples. Seasonal differences were found in free-living and particle-associated bacterial communities, but not in sediment (Table 1). Most environmental factors showed greater variation across sites than across seasons, with temperature being the only exception (Supplementary Figure S4).

**Table 1:** PERMANOVA results of bacterial communities in samples from different sampling sites and seasons [*Bellamya aeruginosa* shell (BAS) = 23, *Corbicula fluminea* shell (CFS) = 22, free-living (FL) bacterioplankton = 27, particle-associated (PA) bacterioplankton = 27, and sediment (S) = 27].

Sample types	Site			Season		
	*R* ^2^	*p*	Significance	*R* ^2^	*p*	Significance
BAS	0.5089	0.001	***	0.1053	0.226	ns
CFS	0.2842	0.001	***	0.2009	0.001	***
FL	0.2350	0.001	***	0.4842	0.001	***
PA	0.2358	0.001	***	0.3937	0.001	***
S	0.4738	0.001	***	0.1000	0.133	ns

Spearman’s correlation analysis was performed to assess the relationship between the relative abundance of dominant phyla and environmental factors (Supplementary Figure S5). In the snail shell samples, Nitrospirota exhibited significant positive correlations with Chl-a, conductivity, DOC, turbidity, TN, and TP. Cyanobacteriota were significantly negatively correlated with these same factors. In clamshell samples, Nitrospirota showed significant negative correlations with Chl-a and temperature, whereas Desulfobacterota was positively correlated with Chl-a, turbidity, and TP. No significant correlations were observed between Cyanobacteriota and any environmental factors in the clamshells.

The null model analysis revealed that the assembly of shell microbiota was primarily governed by deterministic processes (|βNTI | ≥ 2), which accounted for 94.9% of the community assembly in snails and 70.6% in clams (Figure 2e).

### Co-occurrence network of shell bacteria

**Figure 5:**
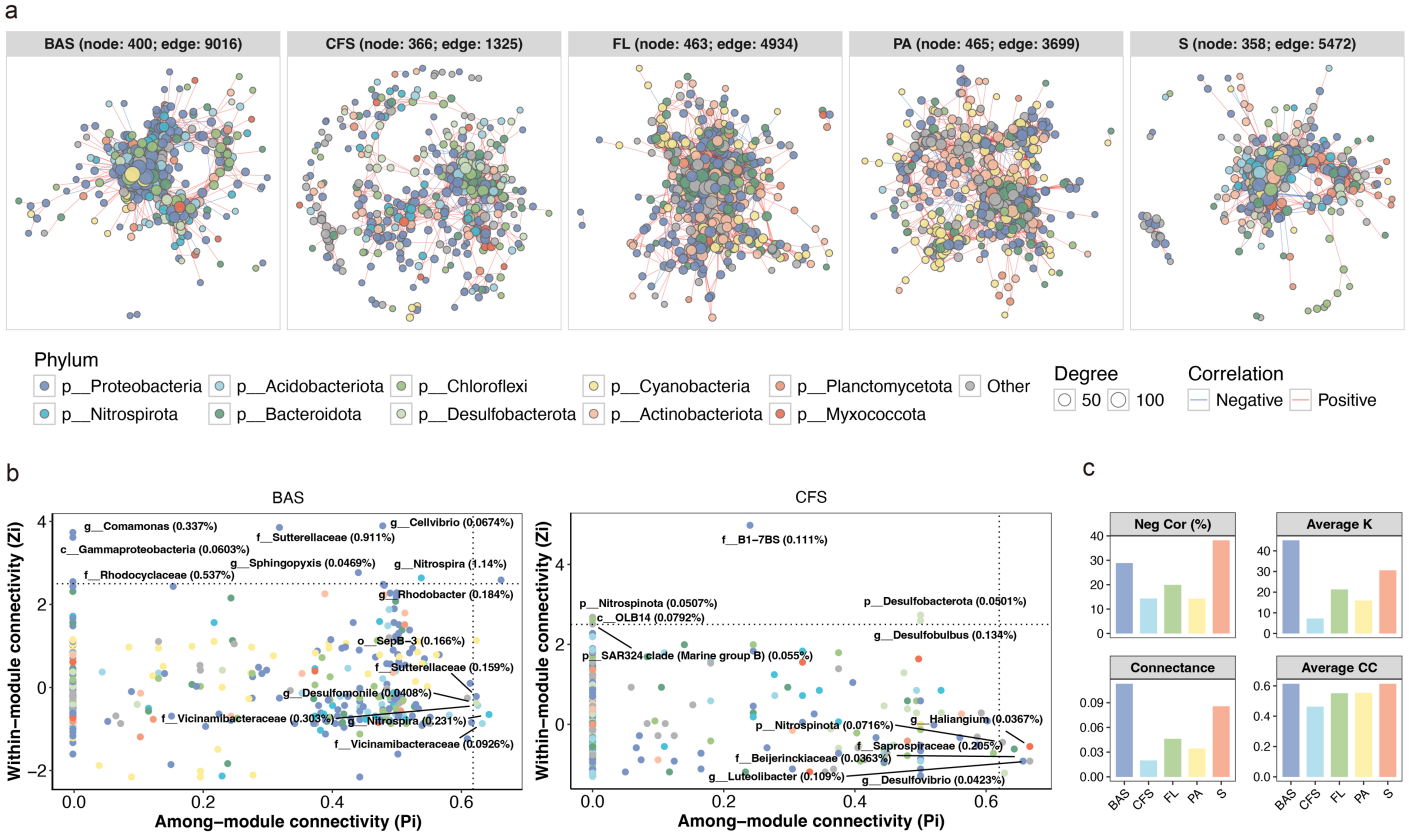
**(a)** Co-occurrence network of *Bellamya aeruginosa* shell (BAS), *Corbicula fluminea* shell (CFS), free-living (FL), and particle-associated (PA) bacterioplankton and sediment (S) samples. Nodes of the network are colored according to the bacterial phylum. The sizes of the nodes reflect the degree of connection. **(b)** Distribution of bacterial ASVs based on their roles in the network of BAS and CFS. The keystone ASVs are labeled with the lowest taxonomy and relative abundance. **(c)** Negative correlations (Neg Cor (%)), average degree (Average K), edge density (Connectance), and mean clustering coefficient (Average CC) of the networks.

Co-occurrence network analysis revealed distinct topological structures across habitats (Figure 5). The network of snail shell-associated bacterial communities consisted of 400 nodes and 9,016 edges, with 28.9% of the correlations being negative. This network included six connectors, seven module hubs, and one network hub identified as keystone taxa, which collectively accounted for 4.55% of the total sequences. The relative abundance of these keystone ASVs ranged from 0.0408 to 1.41%. In contrast, the network for clamshell-associated bacterial communities comprised 366 nodes and 1,326 edges, with 14.3% negative correlations. Here, six connectors and six module hubs were classified as keystone species, representing a total relative abundance of 0.981%, with individual keystone ASV abundances ranging from 0.0363 to 0.205% (Figure 5b). The most tightly interconnected and complex network was associated with snail shell bacterial communities, exhibiting the highest number of nodes and edges, edge density, and average degree, followed by sediment, free-living, particle-associated, and clamshell bacterial communities (Figure 5c).

### Functional prediction of shell bacteria

**Figure 6:**
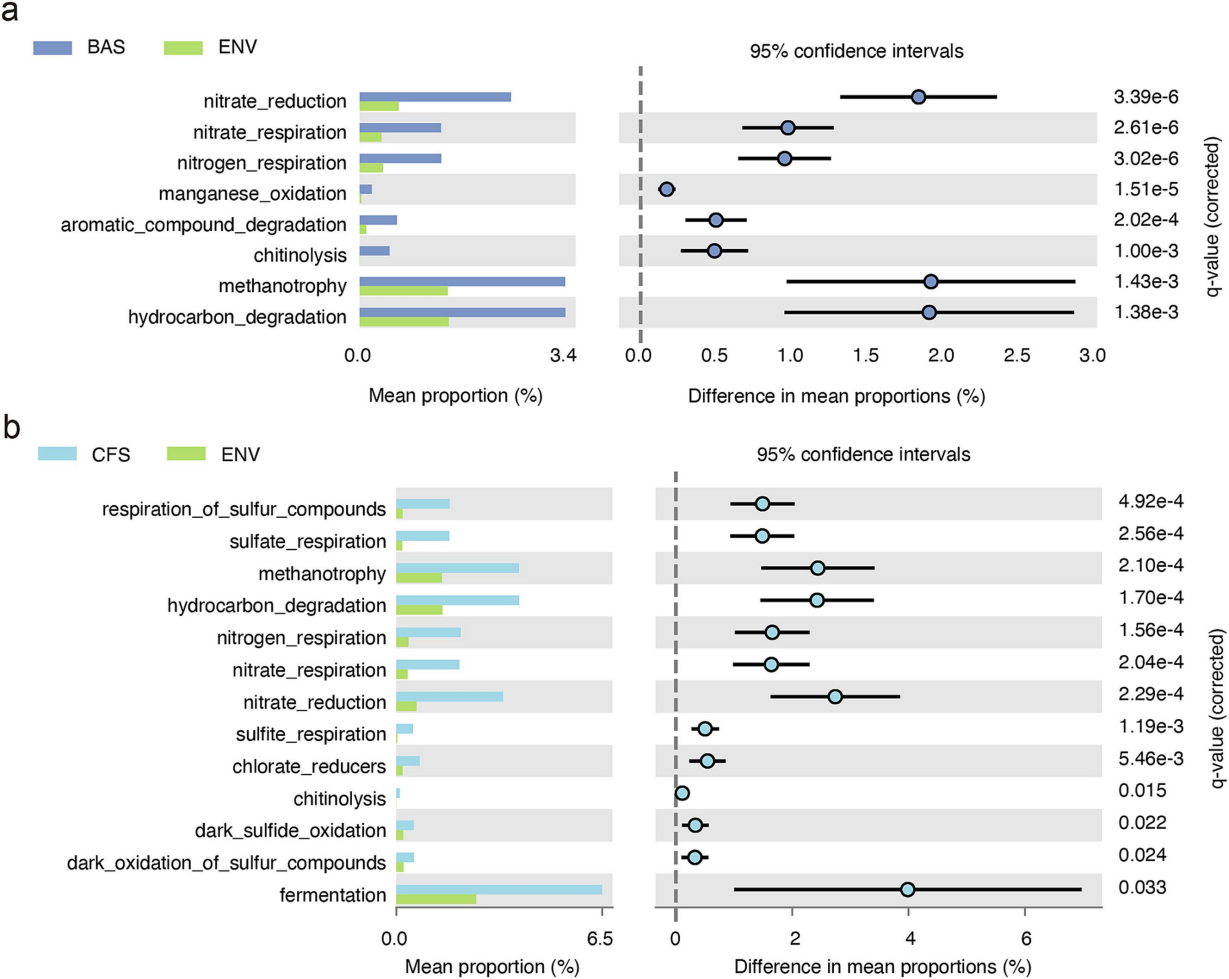
Functional prediction of the bacterial communities significantly enriched in **(a)** *B. aeruginosa* shell (BAS) and **(b)** *C. fluminea* shell (CFS) compared to environmental (ENV) samples (Welch’s *t*-test, 5% CI) (BAS = 23, CFS = 22, and ENV = 81).

According to function prediction by functional annotation of prokaryotic taxa (FAPROTAX), microbial functions, including nitrate reduction, nitrate respiration, nitrogen respiration, manganese oxidation, aromatic compound degradation, chitinolysis, methanotrophy, and hydrocarbon degradation, were significantly enriched in snail shell bacteria in comparison to the environmental microbial communities from the water column and sediment. Respiration of sulfur compounds, sulfate respiration, methanotrophy, hydrocarbon degradation, nitrogen respiration, nitrate respiration, nitrate reduction, sulfite respiration, chlorate reduction, chitinolysis, dark sulfide oxidation, dark oxidation of sulfur compounds, and fermentation were significantly enriched in clamshell bacteria in comparison to the environmental microbial communities from the water column and sediment (Figure 6).

## Discussion

### The potential source and driving factor of shell bacteria

Although mollusk shells are highly exposed to environmental microorganisms such as the water column and sediment, a specific community structure was still observed in the shell bacteria. This suggests that the shells of clams and snails offer a unique ecological niche for specific lake bacteria. In our study, we found that shell bacteria not only represent the bacterial community of the surrounding environment but are also governed by host ecological processes. Source tracking analysis predicted that the potential source, including environmental and tissue samples, contributed up to 89.9 and 97.5% of the bacterial taxa associated with snails and clamshells, respectively, at the genus level. Notably, bacteria associated with the snail shell were mostly derived from sediment, whereas those associated with the clamshell were derived from the host tissue. During the filter-feeding process of a clam, suspended particles are either rejected as mucus-bound pseudofeces or ingested and later excreted as feces (Beninger et al., 1999; Cranford et al., 2011). All biodepositions with tissue microbes, especially mucus-bound pseudofeces, could cling to and colonize the shell, which may explain the strong contribution of the host tissue to the clamshell microbiota. In contrast, deposit-feeding snails maintain closer sediment contact through active gliding and burrowing, resulting in greater sediment-derived microbial colonization of their shells. These findings demonstrate that host ecological processes, particularly feeding behaviors, mediate differential exposure to environmental and tissue-associated microbes, ultimately governing the assembly of shell bacteria.

Our analysis revealed site-specific differences in shell bacteria. However, temporal variation was exclusively observed for clamshells, whereas both snail shells and sediment samples showed no significant seasonal differences. This finding was consistent with the dominant contribution of sediment-derived bacteria to snail shell bacterial communities. Differential exposure to environmental microbes and host life history may contribute to site-specific differences in host-associated bacterial communities (Zhao Z. et al., 2022). Sampling site C, located in a submerged macrophyte-dominated region, maintained a clear-water state. Because of greater light availability, the biomass of periphytic algae tended to accumulate on abiotic and biotic surfaces (Song et al., 2016). Consequently, a higher abundance of cyanobacteria was observed in the shell microbiota at sampling site C than at the other sites. Furthermore, the low mobility of snails provides an ideal surface for algal attachment compared to more mobile clams (Guo et al., 2022). Additionally, snails can climb submerged plants and inhabit areas with greater light exposure, which may explain the higher accumulation of cyanobacteria in their shells (Li et al., 2019). Moreover, we calculated the coefficients of variation (CV) for environmental factors to further assess the spatial and temporal scales of environmental heterogeneity. The results showed that most environmental factors exhibited greater variation across sites than across seasons, a pattern consistent with the microbial community structure. Taken together, these findings suggest that spatial heterogeneity in environmental conditions may play a more prominent role than seasonal variation in influencing the assembly of epibiotic microbial communities in mollusk shells.

Microbial colonization occurs on all natural and artificial surfaces, representing a crucial microbial life mode in aquatic ecosystems. Bacterial communities developing on biotic surfaces differ from those on abiotic surfaces because of differences in selective forces, i.e., host-derived chemical cues (Qian et al., 2022). In our study, *Nitrospira* is the most abundant genus in shell bacteria, which was consistent with the active nitrogen metabolism processes found in the mantle, shell, and surrounding sediment of the bivalve (Svenningsen et al., 2012; Caffrey et al., 2016; Mara et al., 2021). The benthic invertebrate microenvironment is typically rich in ammonium and dissolved organic nitrogen released by host metabolism, potentially promoting the recruitment of nitrifying bacteria to shell surfaces (Boucher and Boucher-Rodoni, 1988; Ray and Fulweiler, 2021). However, *Nitrospira* is also prevalent in various attached growth systems, including rock, plastisphere, tunnel, and water treatment plant biofilms (Pinto et al., 2015; Mehrani et al., 2020; Hou et al., 2024; Su et al., 2024; Kop et al., 2025; Tang et al., 2025). This dominance may stem from *Nitrospira*’s capacity to form microcolonies and biofilms via extracellular polymeric substance (EPS) production (Vijayan et al., 2021). The ecological strategies underlying the dominance of *Nitrospira* in the biofilm remain under investigation; recent evidence suggests that metabolic cooperation, such as cobalamin sharing, may facilitate it to exploit the optimal ecological niche in surface-associated communities (Zhao Y. et al., 2022). Notably, shell bacterial communities from the eutrophic area (sampling sites A and B) harbored higher relative abundances of *Nitrospira* and *Nitrosomonas* compared to the samples from the mesotrophic area of sampling site C. We observed that Nitrospirota in the shell samples exhibited significant positive correlations with Chl-a, conductivity, DOC, turbidity, TN, and TP levels in the water column. Environmental conditions (e.g., nutrient level) and physiological adaptations of nitrifiers may collectively drive the dominance of *Nitrospira* in the shell of mollusks.

### Potential ecological functions of shell microbiota

Mollusks accumulated organic material through feeding, which can be converted by ingested bacteria in the anoxic gut and further released to the environment. It is possible that CH_4,_ NH_4_^+^, and NO^2^-released by the animal and sediment could be further utilized by shell bacteria, which may drive the shell to be a hotspot for the nitrogen cycle in the whole animal, even in the whole water system. In our study, functional prediction revealed that microbial function related to the N and S cycles was significantly enriched in the shell compared to the surrounding environment. Previous evidence on the denitrification measurement of the bivalve oyster shows that the shell microbiota could mediate the nitrogen recycling actively, and the shell of the bivalve can contribute 12–94% of the nitrous oxide release of the whole bivalve individual (Heisterkamp et al., 2013). A higher abundance of the denitrification gene nosZII was observed in oyster shell biofilms than in sediment. Additionally, oyster shell biofilms had a lower (nirS + nirK)/nosZII ratio, indicating a greater capacity for nitrogen removal and limited nitrous oxide release compared to sediments (Filippini et al., 2023). Nitrogen cycling by the abiotic biofilm has gained more attention recently. As discussed above, the dominance of *Nitrospira* is prevalent in attached growth systems. Rate measurement based on stable isotope tracing incubation experiments and multi-omics evidence has confirmed that biofilm is an important nitrifying niche, and the activity of bacteria-mediated nitrification was higher than that of the surrounding seawater (Su et al., 2024; Huang et al., 2025). Furthermore, functions related to sulfur metabolism were also enriched in clamshell bacteria, and many rare ASVs involved in the S cycle were also identified as key species in the network analysis. i.e., species belonging to *Desulfomonile*, *Desulfobulbus*, and *Desulfovibrio*. These rare species can significantly contribute to microbial network stability and ecosystem functioning (Xiong et al., 2020; Chen et al., 2025). Nitrogen and sulfur metabolism were closely coupled in the biofilm system (Nie et al., 2021; Yin et al., 2024). Both sulfate reduction and oxidation processes were observed, implying that a complex gradient of oxygen supply and redox conditions may exist on the shell (Svenningsen et al., 2012). These findings collectively demonstrate that shell-associated microbial communities represent unique ecological niches and potentially serve as important hotspots for biogeochemical transformations in aquatic systems.

## Conclusion

In this study, we selected *B. aeruginosa* and *C. fluminea* as typical snails and clams to explore their shell epiphytic bacteria using 16S amplicon sequencing and functional prediction. We found a deterministic assemblage of shell bacteria governed by host ecological processes, as well as the surrounding environment. We found dominance of the nitrifying genus Nitrospira and enriched functions related to microbial C, N, and S cycles in both shell bacterial communities compared to the surrounding environment, which suggested that shell bacteria are unique ecological niches in the aquatic environment and a hotspot for biogeochemical processes. This study significantly advances our current understanding of host-microbe-environment interactions in benthic ecosystems.

## Data Availability

The datasets presented in this study can be found in online repositories. The names of the repository/repositories and accession number(s) can be found at: https://www.ncbi.nlm.nih.gov/, PRJNA1212329.

## Footnotes

1 http://www.usadellab.org/cms/?page=trimmomatic

2 https://github.com/topics/uclust

3 http://www.arb-silva.de
