# Supplementary material for: Host specificity and uniqueness of shell microbiome in freshwater mollusks
Source: Front Microbiol. 2025 Dec 15;16:1702047. doi: 10.3389/fmicb.2025.1702047 (PMC12747671; doi:10.3389/fmicb.2025.1702047)
Supplement: Supplementary file 1 [file Data_Sheet_1.DOCX]

**Supplementary information**

**Host specificity and uniqueness of shell microbiome in freshwater mollusks**

Zifan Zhao^1^, Zhendu Mao^1^, Dan He^1^, Heng Wang^1^, Qinglong Wu^2, 1, 3, 4,^ *

^1^ Center for Evolution and Conservation Biology, Southern Marine Science and Engineering Guangdong Laboratory (Guangzhou), Guangzhou, People’s Republic of China

^2^ Key Laboratory of Lake and Watershed Science for Water Security, Nanjing Institute of Geography and Limnology, Chinese Academy of Sciences, Nanjing, People’s Republic of China

^3^ Sino-Danish Center for Science and Education, University of Chinese Academy of Sciences, Beijing, People’s Republic of China

^4^ Fuxianhu Station of Deep Lake Research, Chinese Academy of Sciences, Chengjiang, Yunnan Province, People’s Republic of China

The number of words: 578

The number of tables: 2

The number of figures: 5

**Table S1** Samples detail for *Bellamya aeruginosa* shell (BAS), *Corbicula fluminea* shell (CFS), free-living (FL) and particle-associated (PA) bacterioplankton, sediment (S), *Bellamya aeruginosa* tissue (BAT) and *Corbicula fluminea* tissue (CFT) samples

| Season | Site | Sample type | | | | | | |
| --- | --- | --- | --- | --- | --- | --- | --- | --- |
|  |  | BAS | CFS | FL | PA | S | BAT | CFT |
| Autumn | A | 2 | 3 | 3 | 3 | 3 | 3 | 3 |
|  | B | 3 | 3 | 3 | 3 | 3 | 3 | 3 |
|  | C | 3 | 2 | 3 | 3 | 3 | 3 | 3 |
| Winter | A | 3 | 3 | 3 | 3 | 3 | 3 | 3 |
|  | B | 3 | 2 | 3 | 3 | 3 | 3 | 2 |
|  | C | 3 | 3 | 3 | 3 | 3 | 3 | 3 |
| Spring | A | 0 | 3 | 3 | 3 | 3 | 0 | 3 |
|  | B | 3 | 3 | 3 | 3 | 3 | 0 | 3 |
|  | C | 3 | 0 | 3 | 3 | 3 | 0 | 0 |
| Total | | 23 | 22 | 27 | 27 | 27 | 18 | 23 |

**Table S2** Physicochemical characteristics of the water column

| Time | Season | Site | T (°C) | pH | DO (mg/L) | Cond (μS/cm) | NTU | TP (mg/L) | TN (mg/L) | Chl_a (μg/L) |
| --- | --- | --- | --- | --- | --- | --- | --- | --- | --- | --- |
| 2020.10 | Autumn | A | 19.4 | 7.55 | 7.36 | 482 | 167 | 0.0931 | 1.64 | 56.3 |
| 2020.10 | Autumn | B | 19.1 | 8.85 | 17.8 | 357 | 96.5 | 0.0354 | 0.737 | 17.7 |
| 2020.10 | Autumn | C | 19.6 | 9.29 | 10.5 | 305 | 1.93 | 0.0118 | 0.324 | 1.23 |
| 2021.1 | Winter | A | 7.71 | 7.93 | 7.95 | 542 | 84.9 | 0.124 | 2.96 | 32.5 |
| 2021.1 | Winter | B | 7.4 | 8.16 | 12.6 | 427 | 12.8 | 0.0556 | 0.997 | 5.11 |
| 2021.1 | Winter | C | 8.05 | 7.79 | 11.7 | 389 | 23.1 | 0.0225 | 0.296 | 2.08 |
| 2021.5 | Spring | A | 25.3 | 7.51 | 4.87 | 450 | 115 | 0.158 | 1.89 | 152 |
| 2021.5 | Spring | B | 24.4 | 7.96 | 7.23 | 450 | 197 | 0.0764 | 1.22 | 37.2 |
| 2021.5 | Spring | C | 24.7 | 8.74 | 9.14 | 353 | 7.29 | 0.0187 | 0.385 | 3.45 |

**Figure S1** Venn diagrams indicating the distribution of unique and shared bacterial ASVs in the *Bellamya aeruginosa* shell (BAS), *Corbicula fluminea* shell (CFS), free-living (FL) and particle-associated (PA) bacterioplankton and sediment (S) samples. (BAS = 23, CFS = 22, FL = 27, PA = 27, and S = 27)

**Figure S2** Relative abundance of the bacteria associated with *B. aeruginosa* shell (BAS) and *C. fluminea* (CFS) shell samples at the phylum level. Error bars represent standard error. Asterisk above indicates significant differences between *B. aeruginosa* shell and *C. fluminea* shell samples (paired two-sided Wilcoxon rank sum test; ns, not significance, *, p < 0.05, **, p < 0.01, ***, p < 0.001; 95% CI) (BAS = 23 and CFS = 22).

**Figure S3** Bray–Curtis dissimilarity based on bacterial composition of *B. aeruginosa* and *C. fluminea* shell samples visualized using NMDS. (BAS = 23 and CFS = 22).

**Figure S4** Coefficients of variation (CV) for environmental factors across sites and seasons. Points represent individual CV values, with diamonds indicating the mean CV for each group.

**Figure S5** Spearman correlation between the relative abundance of dominant phyla in a) *B. aeruginosa* and b) *C. fluminea* shell samples and environmental factors. (BAS = 23 and CFS = 22).
